# Interferon-α Is the Primary Plasma Type-I IFN in HIV-1 Infection and Correlates with Immune Activation and Disease Markers

**DOI:** 10.1371/journal.pone.0056527

**Published:** 2013-02-20

**Authors:** Gareth A. D. Hardy, Scott Sieg, Benigno Rodriguez, Donald Anthony, Robert Asaad, Wei Jiang, Joseph Mudd, Timothy Schacker, Nicholas T. Funderburg, Heather A. Pilch-Cooper, Robert Debernardo, Ronald L. Rabin, Michael M. Lederman, Clifford V. Harding

**Affiliations:** 1 Department of Pathology, Case Western Reserve University and University Hospitals Case Medical Center, Cleveland, Ohio, United States of America; 2 Department of Medicine, Division of Infectious Diseases, Case Western Reserve University and University Hospitals Case Medical Center, Cleveland, Ohio, United States of America; 3 Center for AIDS Research, Case Western Reserve University and University Hospitals Case Medical Center, Cleveland, Ohio, United States of America; 4 Cleveland Veterans’ Administration Medical Center, Cleveland, Ohio, United States of America; 5 Department of Medicine, University of Minnesota, Minneapolis, Minnesota, United States of America; 6 Department of Obstetrics & Gynecology, Case Western Reserve University and University Hospitals Case Medical Center, Cleveland, Ohio, United States of America; 7 Laboratory of Immunobiochemistry, Center for Biologics Evaluation and Research, United States Food and Drug Administration, Bethesda, Maryland, United States of America; University of Cape Town, South Africa

## Abstract

Type-I interferon (IFN-I) has been increasingly implicated in HIV-1 pathogenesis. Various studies have shown elevated IFN-I and an IFN-I-induced gene and protein expression signature in HIV-1 infection, yet the elevated IFN-I species has not been conclusively identified, its source remains obscure and its role in driving HIV-1 pathogenesis is controversial. We assessed IFN-I species in plasma by ELISAs and bioassay, and we investigated potential sources of IFN-I in blood and lymph node tissue by qRT-PCR. Furthermore, we measured the effect of therapeutic administration of IFNα in HCV-infected subjects to model the effect of IFNα on chronic immune activation. IFN-I bioactivity was significantly increased in plasma of untreated HIV-1-infected subjects relative to uninfected subjects (p = 0.012), and IFNα was the predominant IFN-I subtype correlating with IFN-I bioactivity (r = 0.658, p<0.001). IFNα was not detectable in plasma of subjects receiving anti-retroviral therapy. Elevated expression of IFNα mRNA was limited to lymph node tissue cells, suggesting that peripheral blood leukocytes are not a major source of IFNα in untreated chronic HIV-1 infection. Plasma IFN-I levels correlated inversely with CD4 T cell count (p = 0.003) and positively with levels of plasma HIV-1 RNA and CD38 expression on CD8 T cells (p = 0.009). In hepatitis C virus-infected subjects, treatment with IFN-I and ribavirin increased expression of CD38 on CD8 T cells (p = 0.003). These studies identify IFNα derived from lymph nodes, rather than blood leukocytes, as a possible source of the IFN-I signature that contributes to immune activation in HIV-1 infection.

## Introduction

Type-I interferon (IFN-I) suppresses replication of viruses including HIV-1, induces inflammatory mechanisms, regulates cell survival and promotes maturation and differentiation of antigen presenting cells (APCs) such as dendritic cells (DCs) and monocytes/macrophages [1], [2], [3]. IFN-I is comprised of multiple species, all of which signal through the same heterodimeric receptor (IFN-I-R). In humans, there are twelve subtypes of IFNα, one IFNβ, one IFNω and one IFNκ. IFN-I expression can be induced following activation of innate immune receptors, e.g. Toll-like receptors (TLRs), which recognize microbial structures. IFN-I is produced by numerous cell types. Plasmacytoid DCs (pDCs) produce up to 1000-fold more IFN-I per cell than other cell types [4], [5], [6], but myeloid DCs (mDCs), fibroblasts, epithelial cells and other cells also produce IFN-I. Since these cells are more numerous than pDCs, they may be a significant and under-appreciated source of IFN-I *in vivo*.

Despite indications of an IFN-I signature in HIV-1 infection [7], [8], the predominant IFN-I species that is elevated in HIV-1 infection and its source have not been conclusively determined. While one study reported the presence of an acid-labile IFN-I [9], which was later suggested to be IFNω [10], no subsequent studies have described elevated IFNω levels in the peripheral blood of HIV-1 infected subjects. In 1989, Minagawa et al reported increased peripheral blood IFNβ in HIV-1-infected patients [11], but other IFN-I subtypes were not assessed to provide comparison, and no other studies have since confirmed these results using either updated techniques or characterization of patients according to modern criteria. In 1991, Von Sydow et al reported elevated IFNα in HIV-1-infection [12] which has been supported by others [7]. Although these studies reported elevated IFNα expression in HIV-1 infection [7], [12], the levels of IFNα detected in HIV-1-infected individuals often overlap with the range seen in uninfected donors.

If levels of IFN-I are increased in HIV-1 infection, the cellular source from which they are derived is unclear. Virus-induced IFN-I production by pDCs, a likely source of IFNα, diminishes during the chronic phase of disease. This diminution may be consequence of decreased numbers of circulating pDCs [13], [14], decreased IFN-I production by pDCs, or a combination of both [15], [16], implying that cells other than peripheral blood pDCs are sources for elevated IFN-I levels. Others have suggested that splenic T and B cells may be a source for IFN-I [17]. Uncertainty regarding both the identity and source of elevated IFN-I has limited understanding of the drivers of its expression. As different cell populations produce different IFN-I subtypes, a better understanding of the identity of elevated IFN-I species may guide studies aimed at determining its source and facilitate a greater understanding of its role in HIV-1 infection.

While elevation of IFN-I levels has been difficult to characterize in some studies [7], [12], signatures of IFN-I activity (e.g. expression of IFN-I-stimulated genes, ISGs, and IFN-I-stimulated proteins, ISPs) have been associated with measures of HIV-1 disease pathology [8], [18], [19], [20]. Several studies report elevated plasma levels of interferon-inducible protein-10 (IP-10, also known as CXCL10) in HIV-1 infected patients, even before the onset of symptomatic disease [7], and elevated ISG transcripts have been demonstrated in activated CD4+ T cells from HIV-1 infected subjects [8]. Chronic IFN-I exposure has been implicated in the desensitization of IFN-I signaling in HIV infection, which correlated independently with immune activation [20]. Despite the observation of IFN-I signatures in HIV-1 infection and their correlation with chronic immune activation, the lack of comprehensive direct studies of IFN-I expression in HIV-1 infection leaves the IFN-I signature and the role of IFN-I in HIV-1-infection unexplained.

Our studies were designed to more definitively address IFN-I activity and the presence of IFN-I subtypes in plasma of HIV-1-infected individuals. We report the first comprehensive assessment of plasma levels of all three IFN-I groups (IFNα, IFNβ and IFNω), and we assess correlations with interferon-gamma-induced protein-10 (IP-10) and Myxovirus resistance protein-A (MxA) (two prototypical ISGs/ISPs) in peripheral blood from HIV-1-infected and uninfected subjects. These studies identify IFNα as the predominant IFN-I detectable and elevated in peripheral blood during untreated, chronic HIV-1 infection. In HIV-1-infected subjects who had received successful antiretroviral therapy and achieved durable HIV-1 RNA suppression (<50 copies/ml plasma for >2 years), we found that plasma levels of both IFNα and IP-10 were significantly reduced. In untreated HIV-1 infection, quantitative reverse transcriptase polymerase chain reaction (qRT-PCR) assays revealed that lymph node tissue cells, but not peripheral blood leukocytes, expressed increased levels of IFNα. Expression of the ISG MxA confirmed exposure of both peripheral blood leukocytes and lymph node cells to IFN-I. Furthermore, plasma IFNα levels significantly correlated with plasma HIV-1 RNA levels and markers of immune activation, and inversely correlated with CD4+ T cell decline. In a separate cohort of 7 HCV-infected, HIV-1-uninfected subjects, therapy with pegylated IFNα was associated with sequential increases in markers of immune activation over the course of therapy, suggesting a direct link between IFN-I and immune activation, even in HIV-1-uninfected subjects.

## Results

### IFNα is the Principal IFN-I Detectable in the Peripheral Blood of HIV-1-infected Individuals and Accounts for Peripheral Blood IFN-I Activity

Elevated IFN-I levels are implicated in the pathology of HIV-1 disease by a number of studies, though direct evidence for the presence of specific IFN-I subtypes in the blood of HIV-1-infected individuals remains incomplete. To address these issues we employed ELISA assays for three specific sub-types of IFN-I (IFNα, IFNβ and IFNω) and a bioassay that detects all sub-types of IFN-I (the iLite™ luciferase reporter bioassay, PBL Laboratories) to determine IFN-I activity in peripheral blood plasma from uninfected subjects (n = 28) and subjects with untreated, asymptomatic, chronic HIV-1 infection (n = 61, median CD4 count 405/µl whole blood, median plasma HIV-1 RNA 30,316 copies/ml).

**Figure 1:**
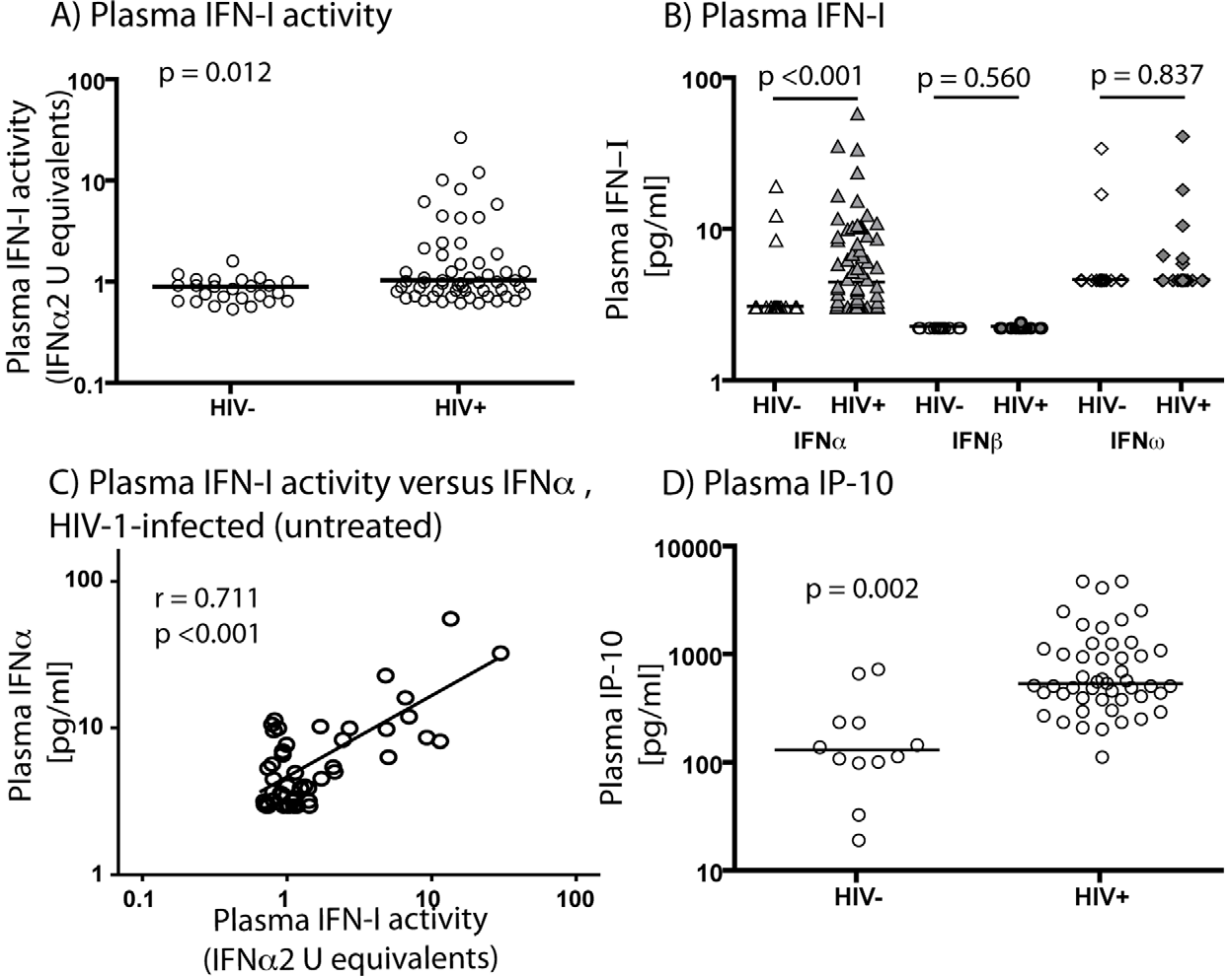
IFNα and its signature are increased in plasma of HIV-1-infected subjects not receiving ART. Plasma IFN-I bioactivity measured by the iLite™ bioassay was increased in plasma of HIV-1-infected subjects in comparison to uninfected subjects (median 1.04 IFNα2 equivalent units for HIV-1-infected subjects, median 0.89 IFNα2 equivalent units for uninfected subjects, p = 0.012) (A). IFNα was increased in plasma of HIV-1-infected subjects in comparison to uninfected subjects (median 4.27 pg/ml for HIV-1-infected subjects, median 3.13 pg/ml for uninfected subjects, p<0.001) (B). IFNβ was not increased in plasma of HIV-1-infected subjects compared to uninfected subjects (median 2.34 pg/ml for HIV-1-infected subjects, median 2.34 pg/ml for uninfected subjects, p = 0.560) (B). IFNω was not increased in plasma of HIV-1-infected subjects compared to uninfected subjects (median 4.69 pg/ml for HIV-1-infected subjects, median 4.69 pg/ml for uninfected subjects, p = 0.837) (B). Plasma IFNα levels were strongly associated with plasma IFN-I bioactivity in HIV-1-infected subjects (r = 0.711, p<0.001) (C). Plasma IP-10 was increased in plasma of HIV-1-infected subjects in comparison to uninfected subjects (median 538.2 pg/ml for HIV-1-infected subjects, median 132.6 pg/ml for uninfected subjects, p = 0.002) (D). Slight variations in sample sizes for different assays occur as results for some subjects were not available.

The magnitude of plasma IFN-I activity (as assessed by the iLite™ assay) showed considerable overlap in the HIV-1-infected and uninfected populations, but a subset of HIV-1-infected individuals showed elevated IFN-I activity, and the difference between the two groups overall was statistically significant (p = 0.012; Fig. 1A). Levels of IFNα, IFNβ and IFNω in the same samples were determined by ELISA. IFNα was the only IFN-I significantly elevated in the plasma of HIV-1-infected subjects compared to uninfected subjects. The median IFNα concentration in plasma of HIV-1-infected subjects (4.27 pg/ml) was significantly greater than that of uninfected subjects (3.13 pg/ml) (p<0.001, Fig. 1B). The percentage of plasma samples with detectable levels (>3.13 pg/ml) of IFNα was 75.9% for HIV-1-infected subjects and 13% for uninfected subjects. In contrast, IFNβ levels were barely detectable in the plasma of either HIV-1-infected or uninfected subjects, even using a recently introduced high-sensitivity assay, and there was no significant difference between the donor groups (p = 0.560; Fig. 1B). IFNω was detected in the plasma of a small number of HIV-1-infected and uninfected subjects, but there was no significant difference between the two groups (p = 0.837; Fig. 1B). In HIV-1-infected subjects, IFNα measured by ELISA strongly correlated with IFNα2 equivalent units by iLite assay (r = 0.711, p<0.001; linear correlation, Fig. 1C). We conclude that the elevated IFN-I activity in plasma of chronically HIV-I-infected subjects is principally IFNα.

### ISG Product IP-10 is Elevated in Plasma of HIV-1-infected Subjects

The chemokine IP-10 (CXCL10) is a well characterized component of the IFN-I signature that is known to be elevated in the plasma of HIV-1-infected subjects [7]. Therefore we investigated whether IP-10 levels were elevated in this cohort, and whether they correlated with IFNα levels. The median level of plasma IP-10 was significantly higher in HIV-1-infected donors (538.2 pg/ml) than in uninfected donors (132.6 pg/ml) (p = 0.002, Fig. 1D). In HIV-1-infected subjects, IP-10 levels were not found to correlate significantly with plasma IFNα (r = -0.050, p = 0.749) or IFN-I activity by iLite™ (r = -0.045, p = 0.773) in HIV-1-infected subjects (data not shown). IP-10 can also be induced by IFNγ, but IFNγ was not detectable in HIV-1-infected subjects in this study who were tested for this cytokine (untreated n = 23, on antiretroviral therapy (ART) n = 8, data not shown). Thus, elevations in IP-10 levels may be an outcome of IFN-I exposure, and the lack of association between plasma IP-10 levels and plasma IFN-I or IFNα levels may reflect unsolved aspects of the role of IFN-I in HIV-1 infection, including the possibility that the IFN-I signature in peripheral blood reflects exposure to IFN-I at extravascular sites, that it involves cooperative signaling by IFN-I-R and other receptors (e.g. Toll-like receptors (TLRs), IFNγ receptor or other pathways directly or indirectly activated by HIV-1), or that IFN-I-induced desensitization of IFN-I signaling [20] complicates the detection of associations between IFN-I and IP-10 in chronic infection.

### Plasma Levels of Both IFNα and IFN-I Signature are Comparable between Uninfected Subjects and HIV-1-infected Subjects Receiving Anti-retroviral Therapy

**Figure 2:**
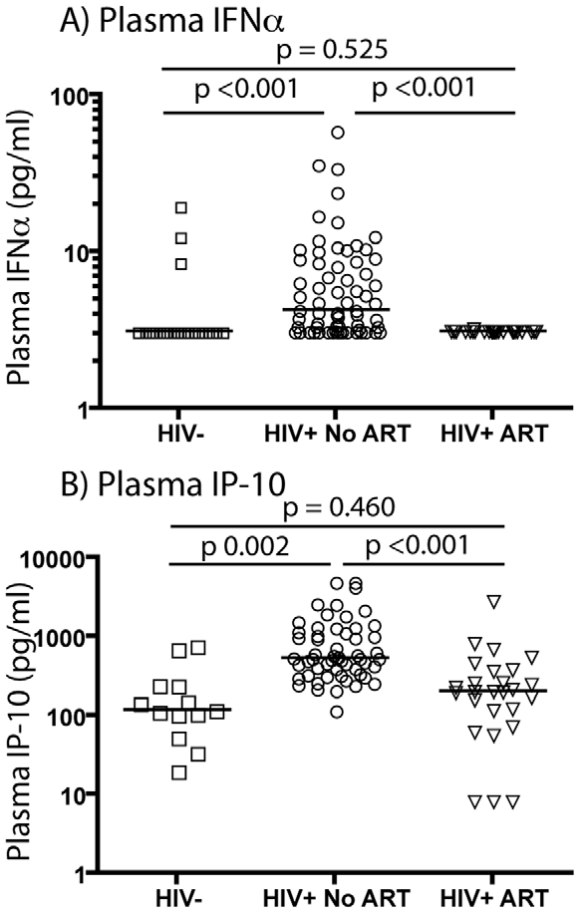
Plasma IFNα and IP-10 levels are comparable between HIV-1 infected subjects on ART and uninfected subjects. IFNα and IP-10 levels were determined in the plasma of 25 individuals with chronic HIV-1 infection who received ART and responded with HIV-1 RNA below assay detection limits (<50 copies/ml) for two years or more. IFNα was detected in plasma from only one such donor, and then at trace levels (A). The difference in plasma IFNα level between HIV-1 infected subjects without ART and HIV-1-infected subjects with ART was highly significant (p<0.001). Plasma IP-10 levels in ART-treated HIV-1-infected donors were comparable to those in uninfected donors (p = 0.460) and were significantly lower in comparison to untreated HIV-1-infected donors (p<0.001) (B).

To determine whether HIV-1-infected subjects receiving effective ART have a distinctive pattern of IFN-I expression, we conducted a cross-sectional assessment of IFNα levels by ELISA in a cohort of 24 chronically HIV-1-infected subjects receiving ART with plasma HIV-1 RNA levels below the limit of detection (<50 copies/ml) for >2 years. Median HIV-1 RNA level was <50 copies/ml plasma and median CD4 count was 531/mm^3^ whole blood. Of these donors, 44% were receiving protease inhibitors, 68% were receiving non-nucleoside reverse-transcriptase inhibitors (NNRTI) and 8% were receiving integrase inhibitors. In all but one treated HIV-1-infected donor (4%), IFNα was below the detection limit of the assay (3.13 pg/ml) (Fig. 2A), and in the one individual with detectable IFNα, the plasma concentration of IFNα (3.24 pg/ml) was barely above the detection limit. The difference in plasma IFNα levels between HIV-1-infected subjects on ART and HIV-1-infected subjects not on ART was highly significant (p<0.001). HIV-1-uninfected subjects showed a pattern similar to that of ART-treated HIV-1-infected subjects (p = 0.525), with only three uninfected subjects having detectable IFNα levels in plasma. Consistent with reduced levels of IFNα in plasma of HIV-1-infected donors receiving ART, we also found that plasma levels of the ISP, IP-10, were similar to those seen in the plasma of uninfected donors (p = 0.460) (Fig. 2B). The difference in plasma IP-10 levels between HIV-1-infected donors receiving ART and those not receiving ART was highly significant (p<0.001). These data suggest that IFN-I expression, and its signature in HIV-1-infected subjects, are driven by mechanisms that are reduced in ART-treated subjects.

### Lymph Nodes, but not Peripheral Blood Leukocytes, are a Source of Elevated Plasma IFNα Levels in HIV-1-infection

**Figure 3:**
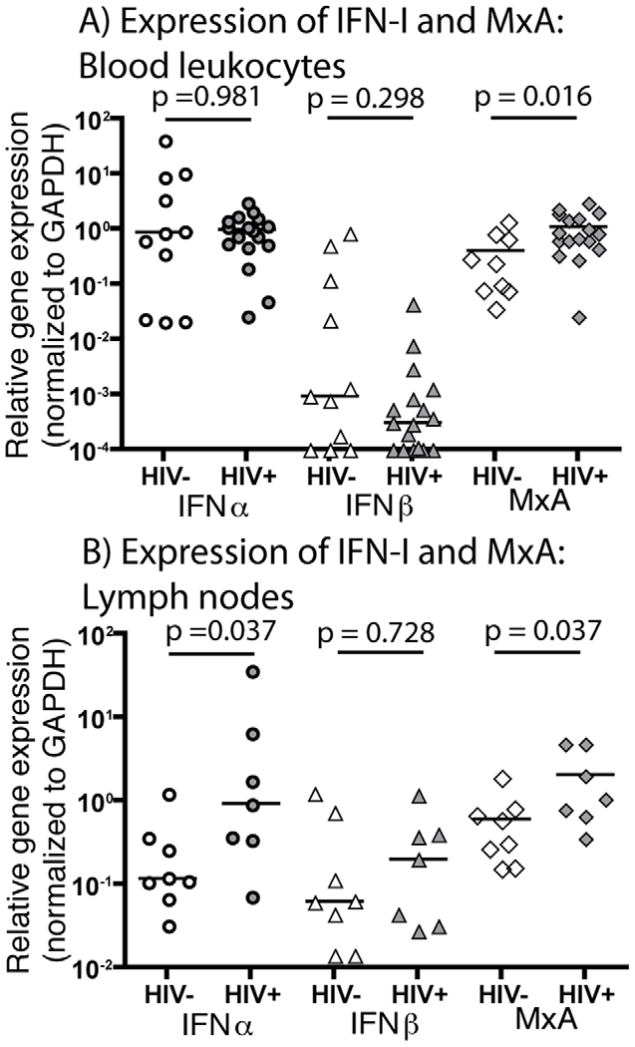
IFNα mRNA expression is elevated in lymph nodes, but not peripheral blood leukocytes, of HIV-1-infected subjects. There was no significant difference in expression of IFNα mRNA in whole blood leukocytes of HIV-1-infected subjects without ART (median relative expression of 0.10) and uninfected subjects (median relative expression of 0.88) (p = 0.981) (A). Similarly, there was no significant difference in IFNβ mRNA expression in whole blood leukocytes (median relative expression of 0.003 for HIV-1-infected subjects, median relative expression of 0.001 for uninfected subjects; p = 0.298) (A). An IFN-I signature was evident in peripheral blood leukocytes, as expression of the ISG MxA was significantly increased in HIV-1-infected subjects without ART compared to uninfected subjects (median relative expression of 0.85 for HIV-1-infected subjects, median relative expression of 0.26 for uninfected subjects; p = 0.016) (A). In contrast, expression of IFNα mRNA in lymph node tissue was significantly elevated in HIV-1-infected subjects without ART (median relative expression of 0.93) relative to uninfected subjects (median relative expression of 0.12) (p = 0.037) (B). There was no statistically significant difference in IFNβ mRNA expression between the two donor groups (median relative expression of 0.20 for HIV-1-infected subjects, median relative expression of 0.06 for uninfected subjects; p = 0.728) (B). Expression of the ISG MxA was significantly increased in lymph node tissue from HIV-1-infected subjects compared to uninfected subjects (median relative expression of 1.05 for HIV-1-infected subjects vs. 0.45 for uninfected subjects; p = 0.037) (B).

To investigate whether peripheral blood leukocytes are a source of IFN-I in HIV-1 infection, we used qRT-PCR to assess expression of IFNα and IFNβ mRNA in leukocytes derived from whole blood (without *in vitro* culture) in order to minimize sample manipulation prior to RNA extraction. Peripheral blood leukocytes from untreated HIV-1-infected subjects did not express higher levels of IFNα or IFNβ mRNA than those from uninfected subjects (p = 0.981 and p = 0.298 respectively, Fig. 3A), suggesting that peripheral blood leukocytes are not the source of elevated IFNα in HIV-1 infection. We next assessed expression of both IFNα and IFNβ mRNA in homogenized lymph node biopsies of HIV-1-infected (untreated) subjects or uninfected subjects. Expression of IFNα was significantly increased in lymph node homogenates of HIV-1-infected donors compared to uninfected donors (p = 0.037), but IFNβ was not significantly increased (p = 0.0728) (Fig. 3B). Although we could not find evidence that peripheral blood leukocytes are a source of elevated IFN-I, we considered ISG expression as evidence for exposure of peripheral blood leukocytes to IFN-I derived from other cells. We investigated the expression of mRNA for the ISG MxA in peripheral blood leukocytes and lymph nodes of uninfected subjects and untreated, chronically HIV-1-infected subjects by qRT-PCR. MxA mRNA expression in peripheral blood leukocytes of HIV-1-infected subjects was significantly greater than that of peripheral blood leukocytes of uninfected subjects (p = 0.016; Fig. 3A). MxA mRNA expression in lymph node cells of HIV-1-infected subjects was also significantly greater than those of uninfected subjects (p = 0.037; Fig. 3B). We conclude that expression of IFNα mRNA is increased in lymph node cells, but not peripheral blood leukocytes, of HIV-1-infected subjects, suggesting that cells in lymphoid tissues, rather than the peripheral blood, are the source of IFN-I that drives IFN-I signature expression in circulating leukocytes.

### Plasma IFN-I Activity and IFNα Correlate Inversely with Peripheral Blood CD4+ T Cell Count and Directly with Plasma HIV-1 Levels and the Activation Marker CD38 on Memory (CD45RO+) CD8+ T Cells

**Figure 4:**
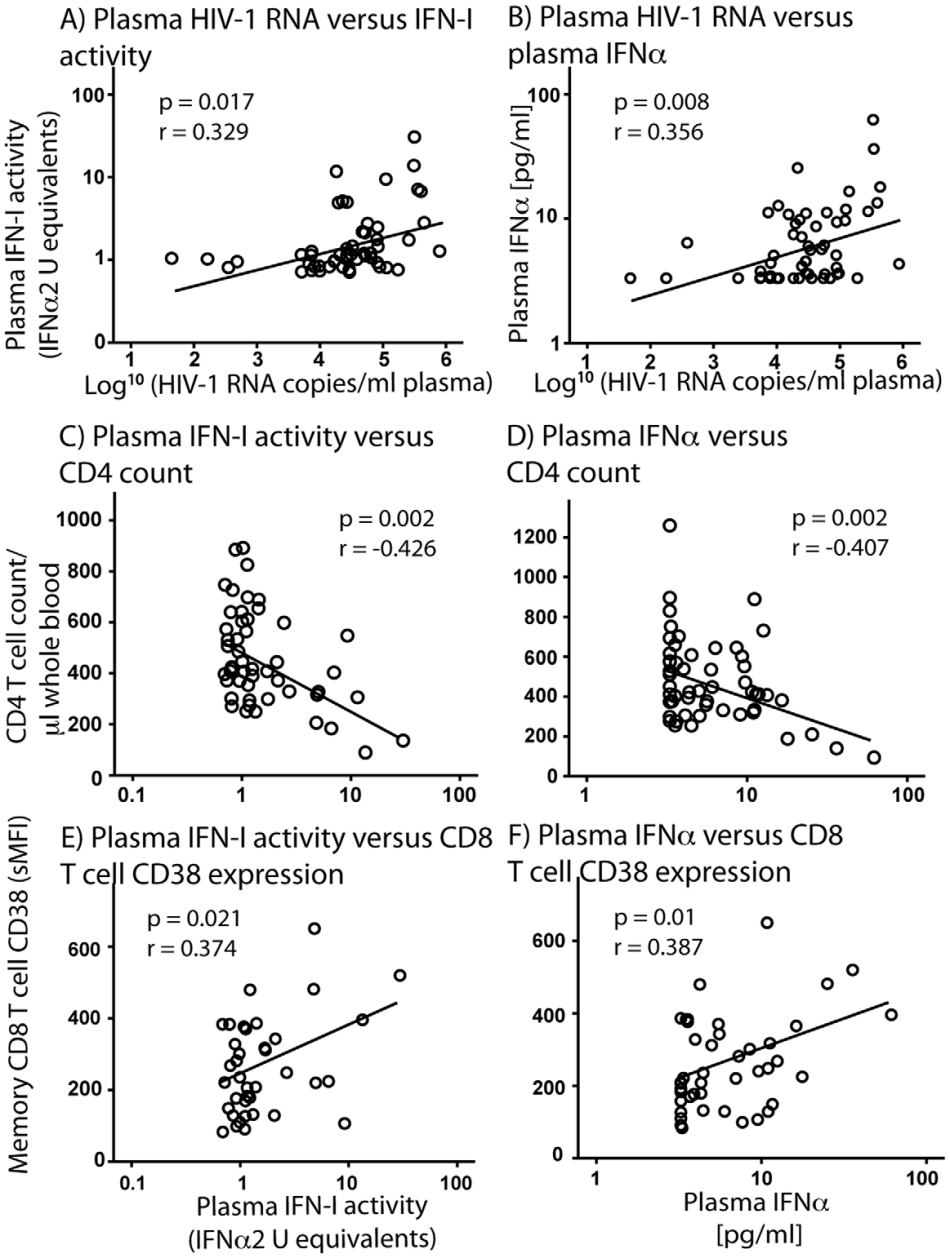
Plasma IFNα and IFN-I bioactivity are associated with plasma HIV-1 RNA levels, CD4 T cell count and immune activation in untreated subjects with chronic HIV-1-infection. Plasma HIV-1 RNA levels in HIV-1-infected subjects without ART correlated positively with plasma IFN-I bioactivity (r = 0.329, p = 0.017) (A), as well as with plasma IFNα (r = 0.356, p = 0.008) (B). Absolute CD4 T cell count in these subjects correlated inversely with plasma IFN-I bioactivity (r = -0.426, p = 0.002) (C) and plasma IFNα (r = -0.407, p = 0.002) (D). Expression of CD38 by memory (CD45RO+) CD8 T cells in these subjects correlated positively with plasma IFN-I bioactivity (r = 0.374, p = 0.021) (E) and with plasma IFNα (r = 0.387, p = 0.01) (F).

As there is considerable variation in IFN-I activity and the IFNα level in plasma of HIV-1-infected subjects, we investigated whether these indices correlate with markers of disease progression. For untreated, chronically HIV-1-infected subjects, plasma IFN-I activity was significantly associated with plasma HIV-1 RNA levels (r = 0.329, p = 0.017; Fig. 4A). Correspondingly, plasma IFNα also correlated with plasma levels of HIV-1 RNA (r = 0.356, p = 0.008; Fig. 4B). In addition, absolute CD4+ T cell count in peripheral blood of chronically HIV-1-infected subjects was inversely correlated with plasma IFN-I activity (r = -0.426, p = 0.002), i.e. lower CD4+ T cell counts are associated with higher plasma IFN-I levels (Fig. 4C). Absolute CD4+ T cell count also correlated inversely with plasma IFNα levels (r = -0.407, p = 0.002; Fig. 4C). Furthermore, immune activation in HIV-1-infected subjects, as determined by the specific MFI of CD38 expression on memory (CD45RO+) CD8+ T cells, was directly associated with plasma IFN-I activity (r = 0.374, p = 0.021; Fig. 4E) and plasma IFNα levels (r = 0.387, p = 0.01; Fig. 4F). These data suggest that elevated IFN-I levels in HIV-1 infection are related to CD4+ T cell decline, HIV-1 RNA levels and chronic immune activation, important features of HIV-1 infection that are closely linked [21].

### Therapeutic Administration of Type-I interferon in HCV-infected Subjects Induces CD38 Expression on Memory CD8 T Cells

The relationship between plasma IFN-I levels and CD38 expression on memory CD8 T cells suggests that immune activation may result from increased IFN-I levels. In order to assess whether IFNα has a direct impact on markers of immune activation *in vivo*, we sought to assess the effects of administration of exogenous IFNα on CD38 expression by memory CD8 T cells *in vivo*. We assessed this effect in HCV-infected subjects rather than HIV-1-infected subjects, since such studies in HIV-1-infected subjects may be complicated by high pre-existing expression of CD38 (see Discussion) [22]. Therefore, the purpose of this study in HCV-infected subjects was to model the effect of IFNα on T cell activation *in vivo*, rather than to assess effects on treatment outcome for HCV infection.

**Figure 5:**
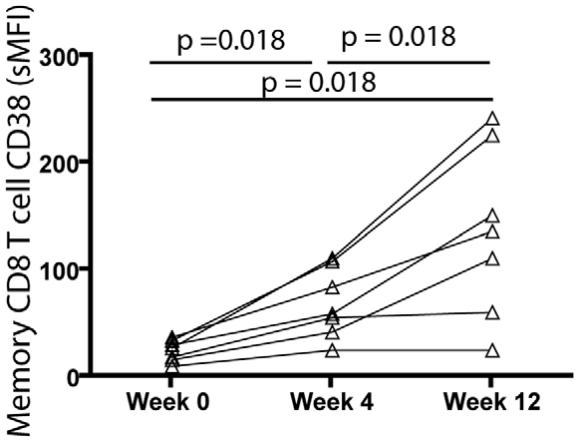
Therapeutic administration of IFNα induces increased expression of the activation marker CD38 on memory CD8 T cells in HCV-infected subjects. A longitudinal study was conducted to assess CD38 expression on memory (CD45RO+) CD8 T cells. Seven HCV-infected (HIV-1-uninfected) subjects were studied immediately prior to initiation of therapy with s.c. pegylated IFNα and oral ribavirin, and after 4 and 12 weeks of therapy. CD38-specific MFI (MFI for CD38 minus MFI for IgG1 isotype control) increased on memory CD8 T cells in all seven treated subjects at 4 weeks of therapy (p = 0.018). A further increase in CD38 was observed in five of seven subjects by week 12 of therapy (p = 0.018). The difference in CD38 expression between week 0 (prior to therapy) and week 12 was highly significant (p = 0.018).

We assessed the expression of CD38 on memory CD8 T cells in a longitudinal study of seven HCV-infected, HIV-1 uninfected subjects who received therapy with pegylated IFNα and ribavirin. Prior to IFNα therapy, CD38 levels were substantially lower in HCV-infected subjects (median specific mean fluorescence intensity (sMFI) for CD38∶27.0) than in the untreated HIV-1 infected cohort studied in Fig. 4 (median sMFI for CD38∶228) (p<0.001). Therapy with pegylated IFNα significantly increased CD38 expression by memory (CD45RO+) CD8 T cells after 4 weeks of treatment (median sMFI for CD38∶59.0, p = 0.018, Fig. 5), at which point CD38 expression increased in all seven donors. By twelve weeks of IFNα therapy there was a further significant increase in CD38 expression by memory (CD45RO+) CD8 T cells (median sMFI for CD38∶136, p = 0.018, Fig. 5). Levels of CD38 expression on memory (CD45RO+) CD8 T cells at twelve weeks were also significantly greater than they were at four weeks (p = 0.018). We conclude that therapeutic IFNα administration substantially increases expression of the activation marker CD38 by memory (CD45RO+) CD8 T cells in subjects with chronic HCV infection. This observation, coupled with the observed correlation between plasma IFNα and CD38 expression on memory (CD45RO) CD8 T cells in HIV-1-infection, suggests that IFN-I may be a causal driver of immune activation (as determined by CD38 expression).

## Discussion

Elevated IFN-I levels are implicated in the pathology of HIV-1 and SIV infection by a number of studies [7], [8], [23], which include observations of increased ISG transcript expression in activated CD4+ T cells of HIV-1-infected subjects [8]. However, most studies have shown significant overlap in IFNα levels between HIV-1-infected and uninfected subjects, and it has remained unclear which of the major IFN-I sub-groups are specifically responsible for the substantive IFN-I signature observed in HIV-1-infected subjects.

In this study, we demonstrate significant elevations in IFN-I bioactivity in the peripheral blood of untreated, chronically HIV-1-infected individuals that are specifically attributable to IFNα, and not to IFNβ or IFNω. In addition, we show that IFN-I levels correlated inversely with CD4+ T cell count and positively with CD8+ T cell activation as determined by CD38 expression on memory CD8 cells. Elevated levels of IFN-I in patients with AIDS were reported as early as 1982 [9] when an acid-labile leukocyte interferon was described (later suggested to be IFNω [10]). Other studies reported increased levels of IFNβ in the peripheral blood of HIV-1-infected patients [11]. Since then, others have found increased levels of IFN-I during the course of chronic HIV-1 infection [12], though the IFN-I sub-types involved have not previously been clearly defined. We have definitively characterized the elevated IFN-I in chronic HIV-1 infection as IFNα. This observation may help to guide investigations of the source for IFN-I in HIV-1 infection and the molecular signals that drive expression of this cytokine.

Even after sustained suppression of viremia during ART, mechanisms that are thought to drive immune activation in HIV-1 infection may persist [24]. Damage to intestinal mucosa, plasma levels of CD14, and chronic T cell activation (as measured by CD38 expression) are all reduced following ART-mediated viral suppression, but often fail to return to levels seen in uninfected individuals [24], [25], [26], [27]. Therefore, we sought to determine if HIV-1-infected subjects receiving ART have concentrations of plasma IFNαthat are different to those we observed in untreated HIV-1-infected subjects. We show that in subjects receiving ART who had undetectable HIV-1 RNA for greater than 2 years, plasma IFNα and IP-10 levels were comparable to those observed in uninfected subjects. These data suggest that expression of IFNα may be driven directly by HIV-1 viremia, or correlates thereof (e.g. viral replication), or indirectly by other mechanisms in HIV-1 disease that are attenuated by effective ART. Candidates for factors that induce IFN-I in HIV-1 infection include viral single-stranded RNA, which is recognized by TLR7/8 [28], [29], and bacterial molecules such as LPS, which is elevated in HIV-1 infection [25] and is recognized by TLR4. TLR4 is not expressed by pDCs, which produce more IFNα per cell than other cell types, but TLR4 drives IFN-I expression in other cell types, and the proportion of IFNα that derives from pDCs during chronic HIV-1 infection remains uncertain. Despite the observation that ART-treated subjects had similar plasma IFNα levels than uninfected subjects, ELISA assays may not be able to detect low levels of IFNα or localized production of IFNα. Thus, it is possible that continued IFNα expression may occur even with suppression of HIV-1 replication, and continued production of IFNα at levels too low for detection by standard ELISA assays may play a role in persistent low-level immune activation.

An increased IFN-I signature may be a more sensitive measurement of the presence of IFN-I than assays targeting IFN-I itself. Therefore we assessed expression of the ISP IP-10 in peripheral blood by ELISA and the ISG MxA in whole blood leukocyte and lymph node tissue RNA by qRT-PCR, and we compared their expression by HIV-1-infected subjects and uninfected subjects. Plasma IP-10 levels were increased in HIV-1-infected subjects, suggesting the presence of a cell population sufficiently exposed to IFN-I to produce detectable quantities of this protein in blood. Peripheral blood leukocytes of HIV-1-infected subjects demonstrated significantly increased expression of MxA compared to that of uninfected subjects, reflecting increased exposure to IFNα. These results are consistent with observations of strong IFN-I gene expression signatures in blood and lymph nodes during chronic SIV infection of rhesus macaques, a non-human primate model of pathogenic lentiviral infection [30], [31]. Measurement of ISG and ISP levels in peripheral blood, i.e. IFN-I signature, may be more sensitive for assessing IFNα exposure than direct measurement of IFNα itself, perhaps because there is inherent amplification in ISG expression.

Although we detected IFNα and its signature in plasma and blood leukocytes, we did not find evidence for increased IFNα mRNA expression in peripheral blood leukocytes of HIV-1-infected subjects. In contrast, IFNα mRNA expression was significantly increased in lymph node tissues of HIV-1-infected subjects, compared to those of uninfected subjects. These observations suggest that the major sources of IFNα in HIV-1 infection are cell populations other than peripheral blood leukocytes. Lymph node cells represent one such source; other tissue sites may also be sources of the excess IFNα responsible for the substantial IFN-I signatures seen in HIV-1 infection. Such sites should be investigated in future studies and may include gastro-intestinal mucosa, liver, vascular endothelium, and bone marrow.

While our data clearly establish IFNα as the primary plasma IFN-I in HIV-1 infection, the expression of IFN-I subspecies in other tissue sites is less explored. Our studies of lymph nodes indicate that mRNA is elevated for IFNα, but not IFNβ, in HIV-1-infected subjects relative to uninfected subjects. This parallels our findings in plasma and suggests that IFNα is the primary IFN-I elevated in lymph nodes in HIV-1 infection, although our lymph node studies do not include data on IFN-I protein or on other IFN-I subspecies, such as IFNω, which we assessed in plasma. In addition, we cannot exclude the possibility of unknown subspecies IFN-I at other tissue sites that have not been studied. Since other IFN-I subspecies are also predicted to induce IFN-I signature (MxA and IP-10), it remains possible that IFN-I subspecies other than IFNα contribute to the IFN-I signature at some sites in HIV-1 infection. In the future it will be useful to study ISG responses as well as IFN-I expression in lymph nodes and other tissues, in HIV-1 infected subjects both before and after ART.

Chronic immune activation is a hallmark of HIV-1 infection and is likely to play a pivotal role in CD4+ T cell depletion during the course of HIV-1 infection [21], [32]. Expression of the marker CD38 on memory CD8+ T cells is a marker of immune activation that may have greater prognostic power for disease progression than HIV-1 RNA levels in plasma or CD4+ T cell count [33]. This relationship is strengthened when excluding naive T cells that constitutively express CD38 [34]. Our data show for the first time a positive association between ex vivo T cell activation (CD38 expression) and both plasma IFN-I activity and IFNα levels in HIV-1 infection. In addition we show that therapeutic administration of pegylated IFNα resulted in significant increases in CD38 expression on memory CD8 T cells of HCV-infected subjects. In an earlier small study of HIV-1 infected persons, administration of pegylated IFNα increased the proportion of T cells that expressed CD38 despite a decrease in plasma HIV RNA levels [22], though the high background CD38 levels in these donors may have reduced the magnitude of CD38 induction by exogenous IFNα. For this reason, we assessed the effect of IFNα administration on CD38 expression in HCV-infected subjects, who do not manifest the elevated CD38 levels that are seen in HIV-1 infection. These results confirm *in vivo* the observations of Rodriguez *et al* who demonstrated the *in vitro* induction of CD38 on CD8 T cells by IFNα [35]. Thus, IFNα may be an important determinant of immune activation in HIV-1 infection.

In summary, our results indicate that IFNα is an important mediator of immune activation and pathology in HIV-1 infection, but is attenuated in ART-treated subjects with suppressed HIV-1 replication. Further investigations are needed to determine the specific molecular signals that induce its expression and its role in the persistent chronic immune activation seen in some long-term ART-treated subjects.

## Materials and Methods

### Study Subjects and Cell Samples

All studies were approved by the University Hospitals Case Medical Center Institutional Review Board and the Cleveland VA Medical Center Institutional Review Board. Written informed consent was obtained from all subjects. For cross-sectional studies of IFN-I and IFN-I signature during untreated chronic HIV-1 infection, participants had not received antiretroviral therapy (ART) or any immunotherapy in the previous year and with HIV-1 RNA load >1,000 copies/ml plasma. Subjects receiving ART had continuous HIV-1 RNA load <50 copies/ml plasma for >2 years and no immunotherapy in the previous two years. Sixty-five untreated HIV-1-infected subjects, twenty-four treated HIV-1-infected subjects, and twenty-seven uninfected subjects were recruited at University Hospitals Case Medical Center, Cleveland, Ohio and donated 30 ml of blood collected into lithium heparin tubes on one occasion each for cross sectional investigations of peripheral blood leukocytes, mononuclear cells (PBMCs) and plasma. Plasma was separated from each sample and stored at −80°C. PBMCs were isolated by differential density centrifugation with endotoxin-free Ficoll-paque™ (GE Healthcare Bio-Sciences, Pittsburgh, PA, USA) for flow-cytometric determination of T cell activation markers. For cross sectional investigations of lymph node IFN-I and MxA levels, pelvic lymph nodes were obtained from HIV-1-infected (untreated) or uninfected donors during surgeries at University Hospitals Case Medical Center, Cleveland, USA; HIV-1-infected individuals had mean whole blood CD4 count of 514 cells/µl whole blood (range 386–653) and mean HIV-1- RNA plasma of 7,465 copies/ml (range 1,189–17,960). Biopsy tissue was placed immediately into RNAlater (Qiagen, Valencia, CA, USA) at 4°C for 12–16 h before freezing in liquid nitrogen. Longitudinal analyses of T cell activation markers were conducted using PBMCs from seven HCV-infected, HIV-1 uninfected subjects initiating parenteral pegylated IFNα and oral ribavirin therapy at the Veterans Administration Hospital, Cleveland, Ohio, USA. These subjects were positive for HCV serum antibody for >6 months and had detectable plasma HCV RNA; they received standard of care therapy with 180 µg pegylated IFNα2a s.c. weekly for 48 weeks in combination with 600 mg ribavirin p.o., b.i.d. Activation markers were assessed at day 0 of therapy and at week 4 and week 12 only.

### Flow Cytometry

PBMCs were stained with the following murine anti-human monoclonal antibodies: anti-CD8-PerCp, anti-CD3-APC, anti-CD45RO-FITC and anti-CD38-PE (All from BD, San Jose, CA, USA). Stained cells were washed in PBS/0.05% sodium azide, fixed in 1% formaldehyde and analyzed with a four-color FACS Caliber flow cytometer and Cell Quest™ software (BD) with an acquisition threshold of 20,000 gated events.

### Quantitative Real-time RT-PCR (qRT-PCR)

RNA was prepared from whole blood and lymph node biopsies for qRT-PCR analysis. Thawed lymph nodes were immediately placed in RLT buffer before homogenization using a tissue homogenizer (Fisher Scientific, Pittsburg, PA, USA). Whole blood lysates were prepared using the QIAamp RNA Blood Minikit (Qiagen). Red blood cells were lysed and remaining leukocytes were placed into RLT lysis buffer. Lymph node and whole blood lysates were then passed through QIAshredder columns (Qiagen) for final homogenization, and mRNA was extracted following on-column DNase digestion using the RNeasy Plus Kit (Qiagen) and stored in RNase-free sterile water at -80°C.

Concentration of mRNA was determined by optical density, and cDNA was reverse-transcribed from mRNA using the oligo(dT)-primer based Superscript II First-Strand Synthesis kit (Invitrogen). Expression of mRNA for IFNα, IFNβ, MxA and glyceraldehyde 3-phosphate dehydrogenase (GAPDH) was assessed by real-time qRT-PCR carried out in triplicate with an iCycler (Bio-Rad, Hercules, CA) using SYBR green detection master mix (Abgene, Rockford, IL). Absolute quantities of mRNA product were determined from a standard curve of serial dilutions of known quantities of each specific amplicon. All results were normalized for GAPDH expression. Primers for GAPDH and MxA were designed according to the NCBI sequences for GAPDH (accession number NM_002046) and MxA (accession number M30817) using Invitrogen primer design web-based software. Sequence specificities were confirmed by BLAST search. Primer pairs were as follows: GAPDH (sense 5′-GACCTGACCTGCCGTCTA-3′; antisense, 5′GTTGCTGTAGCCAAATTCGTT-3′) and MxA (sense, 5′- AGAAGGAGCTGGAAGAAG-3′; antisense, 5′-CTGGAGCATGAAGAACTG-3′). Primers for IFNα (designed for universal amplification of all IFNα sub-types) and IFNβ were used as reported [36]. Primer pairs were as follows: IFNα (sense 5′-GTACTGCAGAATCTCTCCTTTCTCCTG-3′; antisense 5′-GTGTCTAGATCTGACAACCTCCCAGGCACA-3′) and IFNβ (sense 5′-TTGTGCTTCTCCACTACAGC-3′; antisense 5′- CTGTAAGTCTGTTAATGAAG-3′).

### ELISA Assays for IFNα, IFNβ, IFNω and IP-10

ELISA assays were used to determine plasma concentrations of IFNα, β and ω (PBL Laboratories, Piscataway, NJ) and IP-10 (RayBiotech, Norcross, GA). The IFNα assay detected all 12 human IFNα subtypes. The lower limit of detection by ELISA was found to be 3.13 pg/ml for IFNα, 2.34 pg/ml for IFNβ; 4.96 pg/ml for IFNω, and 8.00pg/ml for IP-10. IFN-I or IP-10 standards or plasma were added to pre-coated ELISA wells in a 96-well plate and incubated for 1 h at room temperature. The plates were washed, incubated with biotinylated anti-IFN antibody for 1 h or anti-IP-10 for 2.5 hrs, washed, incubated with streptavidin-horseradish peroxidase for 1 h, washed and incubated with tetramethyl-benzidine substrate for 15 min (all incubations at room temperature). The reaction was terminated with sulphuric acid. Optical density was measured at 450 nm within 5 min using a Bio-Rad model 680 microplate reader.

### Bioassay for IFN-I Activity

Plasma IFN-I activity was measured using a luciferase-based bioassay, the iLite™ IFNα kit (PBL Laboratories). The iLite™ is a firefly luciferase gene-reporter assay using a pro-monocytic human cell line stably transfected with human IFN-I-R for quantitative measurement of biologically active IFN-I levels. Cells were incubated with plasma for 17 h at 37°C. Lysis reagent and substrate were added, and subsequent bioluminescence intensity, proportional to IFN-I activity, was read with a MicroBeta TriLux luminescence counter (Perkin Elmer, Turku, Finland). Relative light units were converted to IFNα2 equivalent Units/ml of plasma using a standard curve with recombinant IFNα2.

### Statistical Analysis

Conventional measures of central location and dispersion were used to describe the data. Pairs of variables were compared with the Mann-Whitney U test. Spearman’s correlation test was used to explore associations between pairs of continuous variables. Repeated measures on the same subjects were analyzed by fitting linear mixed effects models, allowing for both random intercepts and random slopes. Analyses were performed using SPSS, v. 20 (IBM Corp., Armonk, NJ) and Stata MP, v. 11 (Stata Corp., College Station, TX) without explicit correction for multiple comparisons. All tests were two-sided, and p-values ≤0.05 were considered statistically significant.
